# Implementation of a Transfer Intervention Procedure (TIP) to improve handovers from hospital to home: interrupted time series analysis

**DOI:** 10.1186/s12913-016-1730-x

**Published:** 2016-09-07

**Authors:** Rosanne van Seben, Suzanne E. Geerlings, Kim J. M. Verhaegh, Carina G. J. M. Hilders, Bianca M. Buurman

**Affiliations:** 1 Department of Internal Medicine, Division of Geriatric Medicine, Academic Medical Center, PO Box 22660, 1100 DD Amsterdam, The Netherlands; 2 Department of Internal Medicine, Division of Infectious Diseases, Academic Medical Center, PO Box 22660, 1100 DD Amsterdam, The Netherlands; 3 Reinier de Graaf Hospital, Reinier de Graafweg 5, PO Box 5011, 2600 GA Delft, Netherlands; 4 ACHIEVE Centre of Expertise, Faculty of Health, Amsterdam University of Applied Sciences, Tafelbergweg 51, 1105 BD Amsterdam, The Netherlands

**Keywords:** Discharge bundle, Patient handovers, Implementation, Hospitals, Interrupted time series

## Abstract

**Background:**

Accurate and timely patient handovers from hospital to other health care settings are essential in order to provide high quality of care and to ensure patient safety. We aim to investigate the effect of a comprehensive discharge bundle, the Transfer Intervention Procedure (TIP), on the time between discharge and the time when the medical, medication and nursing handovers are sent to the next health care provider. Our goal is to reduce this time to 24 h after hospital discharge. Secondary outcomes are length of hospital stay and unplanned readmission within 30 days rates.

**Methods:**

The current study is set to implement the TIP, a structured discharge process for all patients admitted to the hospital, with the purpose to provide a safe, reliable and accurate discharge process. Eight hospitals in the Netherlands will implement the TIP on one internal medicine and one surgical ward. An interrupted time series (ITS) analysis, with pre-defined pre and post intervention periods, will be conducted. Patients over the age of 18 admitted for more than 48 h to the participating wards are eligible for inclusion. At least 1000 patients will be included in both the pre-implementation and post-implementation group. The primary outcome is the number of medical, medication and nursing handovers being sent within 24 h after discharge. Secondary outcomes are length of hospital stay and unplanned readmission within 30 days. With regard to potential confounders, data will be collected on patient’s characteristics and information regarding the hospitalization. We will use segmented regression methods for analyzing the data, which allows assessing how much TIP changed the outcomes of interest immediately and over time.

**Discussion:**

This study protocol describes the implementation of TIP, which provides the foundation for a safe, reliable and accurate discharge process. If effective, nationwide implementation of the discharge bundle may result from this study protocol.

**Trial Registration:**

Dutch Trial Registry: NTR5951

## Background

Proper patient handovers from hospital to other health care providers are essential in order to provide high quality of care and to ensure patient safety. However, handovers are often delayed [1] and the patient is hardly involved in the discharge process [2, 3]. Besides, communication between secondary and primary care providers is known to be poor [1], clear treatment guidelines for hospital care and post-acute care are missing [4, 5], and healthcare professionals structurally prioritize acute care over post-acute care [6].

Patients are hence discharged with little coordination or follow-up and the transition between hospital and home reflects a vulnerable period. In fact, delays or errors in patient handovers can have serious consequences, including adverse drug events and readmissions within 30-days post-discharge [7].

Similar to the Unites States and England, attention is growing to reorganize and improve the discharge process in the Netherlands. Hospital stays are becoming shorter and an increasing numbers of older patients and chronically ill patients with chronic diseases and/or comorbidity require coordinated and continuous care [8].

Several studies have shown a positive effect, e.g. on readmission rates, of transitional care interventions, which often comprise a bundle of interventions for patients discharged from hospital to their home [9–11]. Yet, these are often comprehensive tailor-made interventions for high-risk patient populations, targeting patient-related factors, whereas cultural and other organizational aspects are important factors that form the basis to ensure the quality and safety of patient handovers for all patients [5]. Therefore, these aspects must be taken into account when interventions are developed. The current study is set to implement the Transfer Intervention Procedure (TIP), a structured discharge process for all adult patients admitted to an internal medicine or surgical ward, with the purpose to provide a safe, reliable and accurate discharge process to all patients.

### Objective

We aim to investigate the effect of a Transfer Intervention Procedure (TIP) on the time between hospital discharge and the time when the medical, medication and nursing handovers are being sent to the next health care provider. Our goal is to reduce this time to 24 h after hospital discharge for all patient handovers. Also, we aim to reduce length of hospital stay and unplanned readmission within 30 days rates.

## Methods

We adhere the SPIRIT guidelines for reporting of trial protocols [12] and all recommended items are addressed in the following paragraphs.

### Study design

**Fig. 1:**
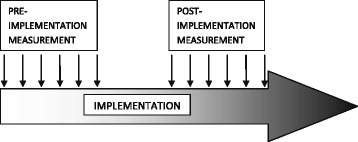
Interrupted Time Series

An interrupted time-series (ITS) study will be conducted from March 2016 until June 2017. There will be six pre-implementation measurements and six post-implementation measurements with 1-month intervals. During the transition period, i.e. 2 months, implementation activities are set up and no measurements will be conducted. Figure 1 provides an illustration of the pre-implementation measurements, implementation period and post-implementation measurements.

An ITS design is the strongest and most commonly used quasi-experimental design to evaluate the impact of an intervention or to measure the effects of a quality improvement when a randomized controlled trial is not feasible or there is no control over the implementation of an intervention [13, 14]. Randomizing implementation of the intervention in the participating hospitals is not possible in the current study, due to practical concerns of the hospitals. Therefore, ITS is chosen as an appropriate and powerful design by which outcomes before and after implementation of the TIP procedure will be compared, while accounting for potential confounders and potential data trends that occur without implementation of the intervention.

### Study setting

This study is embedded in the context of a larger working group of the Dutch Ministry of Health, Welfare and Sport: ‘Addressing Waste in Health Care’. ‘Addressing Waste in Health Care’ is set up in order to reduce inefficiencies in the provision of health care and services and to reduce health care expenditures, for example by reducing the number of preventable hospital readmissions. The implementation of TIP is one of the sub working groups of this larger national program. Eight hospitals (1 university medical center) in the Netherlands will implement the TIP procedure on one of their internal medicine wards and one of their surgical wards.

### The transfer intervention procedure

This study is set to implement the Transfer Intervention Procedure (TIP), which provides the foundation for a safe and reliable discharge process. We aim for a patient handover that is accurate and timely and also transparent to the patient. As previously described [15], a comprehensive discharge checklist may function as key element in standardizing the discharge process. A checklist, containing all elements of the TIP procedure, has to be completed in the patient’s medical record before hospital discharge in order to ensure that the steps will be undertaken. As described elsewhere [16], the TIP procedure was constructed based on focus group meetings with professionals, patient satisfaction surveys and literature. The TIP discharge bundle consists of four elements: 1) determining the discharge date within 48 h after admission and communication of the discharge date with the patient, 2) start with arrangement of required post-discharge care within 48 h after admission, 3) set up patient handover (medical, medication, nurse) and personalized patient discharge letter (PPDL) within 48 h after admission, 4) plan a discharge conversation with the patient to explain information from the PPDL 12 to 24 h before discharge.

### Implementation

The local project leader from the participating hospitals will develop a project plan for implementation of the TIP procedure in their hospital. If required, the study coordinator (RS) will help develop the project plan. All physicians, nurses and medical students involved in patient care on the participating wards will be targeted and motivated to ensure implementation. The plan will include standard items, such as education, reminders and feedback sessions with other participating hospitals, and, if required, local additional items. Table 1 provides an overview of the activities to stimulate implementation of the TIP discharge bundle.

**Table 1:** Description of activities to enhance implementation of TIP

Activity	Aim	Period	For whom	How
Providing information to hospitals about the TIP	Creating awareness Motivating Explanation of TIP procedure Providing background information	At start of implementation period	Project leader Physicians Nurses	Presentation
Patient-education	Creating awareness Patient involvement	During whole implementation period	Patients	Flyers
Results reporting with competitive feedback session	Motivation Awareness of current competition Sharing of experiences with regard to facilitators and barriers of implementation	During whole implementation period	Project leader Physicians Nurses	Presentation Feedback session

### Primary and secondary outcomes

The primary outcome is the number of medical, medication and nursing handovers being sent within 24 h after discharge. Secondary outcomes are length of hospital stay unplanned readmission within 30 days rates.

### Data collection

Variables concerning the primary and secondary outcomes with possible confounders will be collected (see Table 2). These data will be collected from the patient’s medical file and the discharge summary. Castor Electronic Data Capture (EDC) will be used to build electronic Case Report Forms (eCRFs) for save and valid data collection.

**Table 2:** Overview of data collection procedure

	Instrument or Question	Source
Demographical data		Medical record
Age		
Gender		
Socioeconomic status	Postal code	
Marital status		
Living arrangement	e.g. independent, nursing home, sheltered housing.	
Hospitalization		Medical record
Ward	Internal/Surgical	
Index-diagnosis	Acute/Planned	
Length of hospital stay	Time between admission and discharge	
Medical data		Medical record
Presence of polypharmacy	Does the patient uses five or more different medications?	
Comorbidity	Charlson Comorbidity Index [20]	
Number of hospitalizations during six months prior to current hospital stay		
Duration of patient handovers	Time in hours between discharge and the medical, medication and nursing handovers	Medical record
Readmissions	Number of unplanned readmissions within 30 days	Medical record
Emergency department visits	Number of emergency department visits within 30 days	Medical record

### Patients and sample size

All patients over the age of 18 admitted for more than 48 h to one of the 16 participating wards of the eight participating hospitals are eligible for inclusion. The aim is to include at least 22 patients at each time point in all participating hospitals; 11 patients from an internal ward and 11 patients from a surgical ward. With a total of six time points, this will result into at least 65 patients per ward. Hence, we will include at least 1000 patients in the period prior implementation (baseline group) and 1000 patients in the period after the implementation (intervention group). This number is based on the number of hospital beds at the participating wards and feasibility with regard to data collection. We conducted a power analysis with this number of patients, which is based on the findings of a previous study [16]. However, we expect to find a greater effect in the current study, namely a reduction of 78 % in the time of patient handovers being sent to the next health care provider. In a simulation study with 16 wards, each contributing 65 patients, we estimated the power to be approximate 91 % to demonstrate a reduction of 78 % in time until sending the medical discharge letter, assuming that the intraclass correlation coefficient does not exceed 0.05.

### Segmented regression of interrupted time series analysis

We will use segmented regression methods for analyzing the data, which is most commonly used to assess ITS data as it allows assessing how much an intervention changed a certain outcome immediately and over time [13]. We will use segmented regression analysis to assess significance of changes in level and slope of the regression lines before and after implementation of the TIP procedure. We will collect data 6 months prior to implementation of the intervention and 6 months after implementation. We will adjust for autocorrelation, which refers to the serial dependence of outcome measure error terms, in order to avoid underestimating standard errors and overestimated significance of the effects of the TIP procedure [14]. We will consider non-stationary data, taking into account other interventions implemented in the same period or changes in hospitals’ policies.

### Qualitative evaluation of patient satisfaction and facilitators and barriers of implementation

In addition to quantitative data collection, comprehensive qualitative data regarding the implementation of the intervention may provide valuable additional information when an implementation evaluation is conducted [13]. First, in order to evaluate patient satisfaction and the patient’s perspective on the discharge procedure, semi-structured interviews will be conducted with patients shortly after discharge. Question will be asked regarding their experience with the discharge procedure. For example, patients will be asked whether they felt confident about returning home and if they felt they were well informed. Also, their opinion with regard to the discharge conservation and personalized patient discharge letter (PPDL) will be assessed. Post-implementation, RS will interview patients until saturation of the data emerges [17].

Second, one focus group discussion will be conducted with the project leaders of all participating hospitals and eight focus group discussion will be conducted with the project groups of each hospital. These group discussions will be used in order to reflect the social and cultural context of barriers and facilitators to implement the TIP and improve the discharge process in the hospital. The project leaders are, together with their project group, responsible for local implementation of TIP. The project groups consist of the project leader and, for example, the head of the participating ward, a senior nurse and physicians. An experienced moderator (RS) will lead all focus group interviews.

The interviews and focus groups will be recorded, after written consent is obtained from the participants, and transcribed verbatim. Data derived from the interviews with patients and focus groups discussions with professionals will be analyzed iteratively, consisting of a combination of open coding and sensitizing concepts by which constant comparisons are made [18, 19]. MAXQDA Software will be used in order to facilitate the coding process of both the semi-structured interviews with patients and the focus group discussions.

### Validation and process evaluation

In order to assess compliance of the professional with the TIP, we will evaluate the percentages of compliance with regard to process indicators on the checklist, including discharge planning within 48 h, start with arrangement of required aftercare within 48 h (if needed), holding a discharge conversation within 12 to 24 h before discharge and providing a patient letter. Also, in order to determine whether there is any compliance with regard to these process indicators prior to implementation of the TIP procedure, the number of patients for whom these aspects of the TIP procedure were done will be considered.

## Discussion

This study protocol describes the design, implementation and evaluation of the Transfer Intervention Procedure (TIP); a discharge bundle to improve discharge care on an organizational level. We aim for a one hundred percent of medical, medication and nursing handovers being sent within 24 h to the next health care provider. Yet at the same time, professionals should be aware that this does not come at the expense of the content of the patient handovers. To our knowledge this is the first study that investigates the implementation of such a discharge bundle on a large, national-scale, in eight different Dutch hospitals. If effective, nationwide implementation of the discharge bundle may result from this study protocol.
